# RUCAM in Drug and Herb Induced Liver Injury: The Update

**DOI:** 10.3390/ijms17010014

**Published:** 2015-12-24

**Authors:** Gaby Danan, Rolf Teschke

**Affiliations:** 1 Pharmacovigilance Consultancy, rue des Ormeaux, 75020 Paris, France; 2 Department of Internal Medicine II, Division of Gastroenterology and Hepatology, Klinikum Hanau, Academic Teaching Hospital of the Medical Faculty, Goethe University Frankfurt/Main, Frankfurt am Main, D-63450 Hanau, Germany; rolf.teschke@gmx.de

**Keywords:** drug induced liver injury, drug hepatotoxicity, herb induced liver injury, herbal hepatotoxicity, drugs, herbs, dietary supplements, causality assessment, RUCAM, CIOMS

## Abstract

RUCAM (Roussel Uclaf Causality Assessment Method) or its previous synonym CIOMS (Council for International Organizations of Medical Sciences) is a well established tool in common use to quantitatively assess causality in cases of suspected drug induced liver injury (DILI) and herb induced liver injury (HILI). Historical background and the original work confirm the use of RUCAM as single term for future cases, dismissing now the term CIOMS for reasons of simplicity and clarity. RUCAM represents a structured, standardized, validated, and hepatotoxicity specific diagnostic approach that attributes scores to individual key items, providing final quantitative gradings of causality for each suspect drug/herb in a case report. Experts from Europe and the United States had previously established in consensus meetings the first criteria of RUCAM to meet the requirements of clinicians and practitioners in care for their patients with suspected DILI and HILI. RUCAM was completed by additional criteria and validated, assisting to establish the timely diagnosis with a high degree of certainty. In many countries and for more than two decades, physicians, regulatory agencies, case report authors, and pharmaceutical companies successfully applied RUCAM for suspected DILI and HILI. Their practical experience, emerging new data on DILI and HILI characteristics, and few ambiguous questions in domains such alcohol use and exclusions of non-drug causes led to the present update of RUCAM. The aim was to reduce interobserver and intraobserver variability, to provide accurately defined, objective core elements, and to simplify the handling of the items. We now present the update of the well accepted original RUCAM scale and recommend its use for clinical, regulatory, publication, and expert purposes to validly establish causality in cases of suspected DILI and HILI, facilitating a straightforward application and an internationally harmonized approach of causality assessment as a common basic tool.

## 1. Introduction

Liver injury is a complex, challenging disease with multifaceted features common to both drug induced liver injury (DILI) [1,2,3,4,5,6,7,8,9] and herb induced liver injury (HILI) [10,11,12,13,14,15,16,17]. Current hallmarks focus on our understanding and handling of liver injury cases including case definition and phenotype standardization [18]. Genome-wide association studies have identified genetic predisposition as a relevant risk factor for liver injury [19,20]. In particular, human leucocyte antigen (HLA) genotype is a strong risk factor for the development of DILI with a range of drugs, likely involving a drug-peptide complex to T cells, but HLA alleles are only associated with some forms of DILI [19]. Non-HLA genetic risk factors appear to play a contributory role, especially those related to drug metabolism, detoxification, and disposition [19,20]. Involved genes may cause polymorphisms of bioactivation pathways via the cytochrome P450 (CYP) systems (Phase I), detoxification reactions (Phase II), and excretion and transport (Phase III) [20]. For some drugs, even a dual role of HLA and drug metabolism genes is under consideration [19]. Other risk factors of DILI and HILI include preexisting liver disease although still debatable [6,7], comedication [21], drug lipophilicity [22], and high daily doses [23].

Our knowledge of pathogenetic aspects related to DILI and HILI has substantially increased within the past years and decades [1,2,3,4,5,6,7,8,9,10,11,12,13,14,15,16,17,18,19,20,21,22,23], but little if any progress has emerged in the clinical setting to improve diagnostic tools. Despite major efforts worldwide, we are far away from any realistic goal having a valid diagnostic biomarker that may help clinicians to establish a firm diagnosis of DILI and HILI in all suspected cases. This is more than disappointing in face of existing numerous valid clinical biomarkers that enable a clear diagnosis of most liver diseases unrelated to DILI and HILI, for instance by assessing specific antibodies of viral hepatitis, specific immunological parameters in autoimmune liver diseases, or specific parameters in genetic liver diseases [14,15]. In the majority of the DILI and HILI cases, injury is the result of an idiosyncratic reaction at recommended doses [8,14,18]. This leads to only small amounts of toxic metabolites in the liver that are undetectable in the blood as diagnostic biomarkers [14]. Conditions are quite different in the rare liver injury cases of the intrinsic type, which are dose dependent and caused by compounds that are well measurable in the blood [14,24,25,26,27]. Examples are hepatotoxicity cases by overdosed acetaminophen with measurable acetaminophen-cysteine adducts in acute liver failure [24] or the toxic hepatic sinusoidal obstruction syndrome caused by plants containing unsaturated pyrrolizidine alkaloids (PAs) [14,25,26,27], where pyrrole-protein adducts can be assessed in the blood of affected patients [26]. Hepatic microsomal cytochrome P450 is involved in the metabolic activation of PAs to electrophilic pyrrolic metabolites that react with macromolecules such as proteins and lead to the formation of pyrrole-protein adducts [25,26]. The analytical approach detecting these adducts in the blood represents a valuable specific and sensitive diagnostic biomarker [26] that is otherwise rarely available in suspected DILI and HILI cases and complicates the diagnostic work-up.

By far the most annoying flaws during clinical causality assessment of patients with suspected liver injury are alternative diagnoses, as these patients are not provided in time with the appropriate specific therapies, which are substantially different from those of the initial incorrect diagnosis of DILI or HILI [16,28,29,30,31,32]. Missed diagnoses are often described in the literature and could occur at any evaluating level, beginning with the caring physician, continuing among expert groups, and ending during the evaluation by the regulatory agencies [31,32]. These specific problems as well as other confounding variables such as poor data quality, comedication, and vague interpretation of challenge, dechallenge and rechallenge conditions in DILI have early been recognized and led to the development of a new causality assessment method (CAM) for DILI. This was originally named RUCAM (Roussel Uclaf Causality Assessment Method) [8,9] or later also synonymously CIOMS (Council for International Organizations of Medical Sciences) [33,34,35,36]. RUCAM was developed to cope with the shortcomings inherited in causality assessment of DILI [8,9]. It is well validated by cases with positive reexposure tests serving as a gold standard [9]. Most importantly, RUCAM is a means of assigning points for clinical, biochemical, and serologic features as well as search for non-drug causes.

Summing up the points of the criteria gives an overall assessment score that reflects the likelihood that the hepatic injury is due to a specific medication [8]. Details of the structured, hepatotoxicity specific, and quantitative RUCAM were published in 1993 [8,9]. Since then, physicians, expert groups, pharmaceutical companies, and regulatory agencies have a long practical experience with this original RUCAM, which needs actualization and refinement [33]. Few weaknesses have been recognized [33,34,35,36], and some were already considered previously by item actualization and precision [35,36].

In this review article, we present new developments of RUCAM and provide the update for diagnostic criteria that required refinement due to newly established analytical tools and for reasons of accuracy. The aims are also to reduce interobserver and intraobserver variability, to present accurately defined core elements, and to simplify the handling of the items. Now, an internationally harmonized approach of causality assessment for DILI and HILI cases is recommended, applying this updated RUCAM as a common basic tool for clinical, regulatory, publication, and expert purposes.

## 2. Data Sources and Searches

### 2.1. Search Terms

We used the PubMed database to identify publications for the following terms: for RUCAM, and around 6200 hits were provided; for Roussel Uclaf Causality Assessment Method.4350 hits; for CIOMS, 191,000 hits; for Council for International Organizations of Medical Sciences, 7,080,000 hits; for the combination of RUCAM/CIOMS or CIOMS/RUCAM, each 2890 hits; for RUCAM DILI, 11,400 hits; for CIOMS DILI, 6080 hits; for RUCAM HILI, 1680 hits; and for CIOMS HILI, 1900 hits. This search revealed that DILI is more often associated with RUCAM and HILI with CIOMS. Since liver injury relates more often to DILI rather than to HILI, and due to historical background and the original work with its validation, we suggest the term RUCAM should be used solely in the future in connection with both DILI and HILI, dismissing the term CIOMS for reasons of clarity and simplicity.

### 2.2. Data Extraction

We used primarily our large and actualized private scientific archives, which contain original full-length publications relating to RUCAM, CIOMS, DILI, and HILI. In addition, the search for additional publications was extended through the PubMed database. Prior to our analysis, the publications were assessed regarding their scientific and clinical quality. Publications of relevance and good quality were preferred and considered for evaluation. The focus of our search was on publications in English language, but relevant reports of other languages were also considered. The retrieved publications included case reports, case series, and review articles; they were analyzed to assess whether they were appropriate and relevant for the topic of this article. Publications were also manually searched for additional publications not yet identified. The literature search ended on 4 November 2015. The final compilation of evaluated publications consists of original papers, case series, case reports, consensus reports, and review articles. The relevant reports were included in the reference list of this review. Publication of the analyzed reports was commonly between 1977 and 2015, preferentially within the last decade.

## 3. A General View Back to the Original RUCAM

Historically, the development of RUCAM goes back to the late 1980s, partially based on results of international consensus meetings of experts as documented by various reports [37,38,39,40] and reviewed recently [36]. Establishing RUCAM with all core items and specific details was time-consuming and required some years as RUCAM was the first liver-specific causality CAM for DILI ever published worldwide [8,9]. Originally, the criteria of causality assessment and their weight can be traced back to a French method for general drug reaction assessment that was not liver-specific and merely qualitative [37]. This original French method considered criteria under both chronological and clinical aspects. The chronological criteria included three datasets: time to onset of the reaction, the clinical course after cessation or continuation of the drug, and the response to readministration. Answers to these items were combined in a decision table, leading to an overall qualitative chronology score rated as incompatible with, dubious, possible, or suggestive of a drug-induced reaction. The clinical criteria also included three different items: signs and symptoms suggesting the causal role of the drug and/or presence of a risk factor; result of a specific test proving the causal role of the drug; and assessment of non-drug causes [37]. These results were then again combined in a decision table, leading to the clinical assessment as dubious, possible, or suggestive. Finally, chronological and clinical scores were combined, and this resulted in a causality assessment of very likely, likely, dubious, possible, or unlikely [37]. Based on the chronological and clinical criteria of this general and organ-unrelated assessment [37], parts of these qualitative scores have then been adapted specifically for DILI but their initial application was less user-friendly [39].

### 3.1. Nomenclature of RUCAM

Consensus meetings of experts aiming to define terms used in DILI assessment and qualitative criteria based on the French CAM to be applied for DILI have been organized by the French Roussel Uclaf pharmaceutical company since 1985 [9,37]. The results of the meeting published in 1990 were incorporated into a new CAM, which included additional criteria and ascribed weight to each criterion, leading to the name Roussel Uclaf Causality Assessment Method (RUCAM) published in 1993 [8,9]. Its previous alternative name of CIOMS (Council for International Organizations of Medical Sciences) ascribed by some authors applying RUCAM goes back to the fact that the consensus meeting in Paris in 1989 was held under the auspices of the Council for International Organizations of Medical Sciences (CIOMS), directed at establishing definitions and uniform criteria for DILI [40]. These criteria were included in a diagnostic instrument for causality assessment but were fine-tuned, other criteria were added, a score was ascribed to each criterion depending on the type of the liver injury, and finally the method was validated with cases including positive rechallenge as a gold standard. Those who then applied this final scale [8,9] called it either RUCAM or CIOMS in their publications [33,34,35,36]. There was some preference for the term RUCAM in the United States, whereas experts in Europe and the other countries preferred the term CIOMS. Rather than using both terms interchangeable as in the past, we now prefer for the future only one single term, namely RUCAM, as this is also the most quoted method for DILI in the PubMed database.

**Figure 1:**
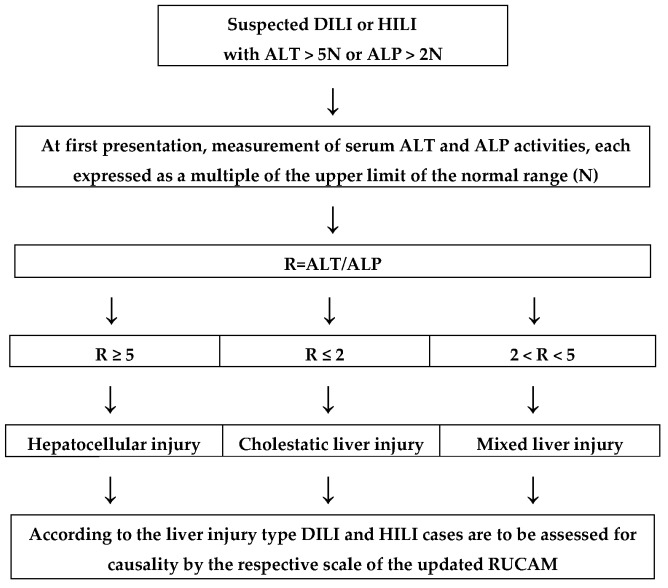
Classification of liver injury required for causality assessment of suspected DILI and HILI cases by the updated RUCAM. Note above: ALP from hepatic origin only. Abbreviations: ALP, alkaline phosphatase; ALT, Alanine aminotransferase; DILI, Drug-induced liver injury; HILI, Herb-induced liver injury; N, Upper limit of normal; R, Ratio; RUCAM, Roussel Uclaf Causality Assessment Method.

Consequently, the improved RUCAM version we now present is the updated RUCAM. Definition and classification of liver injury (Figure 1) and specific operational information (Table 1) are to be considered prior to applying the updated RUCAM. This is designed specifically for either the hepatocellular injury (Table 2) or the cholestatic and mixed liver injury (Table 3). For assessment of future DILI and HILI cases, we also strongly recommend applying solely one of the updated RUCAM versions (Table 2 and Table 3) presented in this review rather than any previous original and otherwise modified version that we now consider obsolete [8,9,33,35,36,38,39,40,41,42,43].

**Table 1:** Specific operational information on the updated RUCAM.

Operational Information on the Updated RUCAM	Ref.
1. RUCAM affords prospective use, since retrospective scoring is less accurate.	[8,15]
2. RUCAM is to be calculated individually for each co-administered product.	[8,15]
3. RUCAM is conceptualized primarily for idiosyncratic, not for intrinsic reactions.	[8]
4. RUCAM excludes cases with onset of hepatic injury before start of product use.	[8]
5. RUCAM is applicable only for acute liver injury, not for preexisting chronic liver disease.	[8]
6. RUCAM cannot correctly assess when ALP is elevated for non-hepatic reasons.	[8]

Abbreviations: ALP, Alkaline phosphatase; RUCAM, Roussel Uclaf Causality Assessment Method.

**Table 2:** Updated RUCAM for the hepatocellular injury of DILI and HILI. The items specifically refer to the hepatocellular injury rather than to the cholestatic or mixed liver injury (shown in Table 3). Abbreviations: ALT, Alanine aminotransferase; AST, Aspartate aminotransferase; CMV, Cytomegalovirus; CT, Computer tomography; DILI, Drug induced liver injury; EBV, Epstein Barr virus; HAV, Hepatitis A virus; HBc, Hepatitis B core; HBsAg, Hepatitis B antigen; HBV, Hepatitis B virus; HCV, Hepatitis C virus; HEV, Hepatitis E virus; HILI, Herb induced liver injury; HSV, Herpes simplex virus; MRC, Magnetic resonance cholangiography; N, upper limit of the normal range; RUCAM, Roussel Uclaf Causality Assessment Method; VZV, Varicella zoster virus. Total score and resulting causality grading: ≤0, excluded; 1–2, unlikely; 3–5, possible; 6–8, probable; and ≥9, highly probable.

Items for Hepatocellular Injury	Score	Result
1. Time to onset from the beginning of the drug/herb		
● 5–90 days (rechallenge: 1–15 days)	+2	□
● <5 or >90 days (rechallenge: >15 days)	+1	□
Alternative: Time to onset from cessation of the drug/herb		
● ≤15 days (except for slowly metabolized chemicals: >15 days)	+1	□
2. Course of ALT after cessation of the drug/herb		
Percentage difference between ALT peak and N		
● Decrease ≥ 50% within 8 days	+3	□
● Decrease ≥ 50% within 30 days	+2	□
● No information or continued drug use	0	□
● Decrease ≥ 50% after the 30th day	0	□
● Decrease < 50% after the 30th day or recurrent increase	−2	□
3. Risk factors		
● Alcohol use (current drinks/d: >2 for women, >3 for men)	+1	□
● Alcohol use (current drinks/d: ≤2 for women, ≤3 for men)	0	□
● Age ≥ 55 years	+1	□
● Age < 55 years	0	□
4. Concomitant drug(s)/herb(s)		
● None or no information	0	□
● Concomitant drug/herb with incompatible time to onset	0	□
● Concomitant drug/herb with compatible or suggestive time to onset	−1	□
● Concomitant drug/herb known as hepatotoxin and with compatible or suggestive time to onset delete marking right side above	−2	□
● Concomitant drug/herb with evidence for its role in this case (positive rechallenge or validated test)	−3	□
5. Search for alternative causes	Tick if negative	Tick if not done
Group I (7 causes)		
● HAV: Anti-HAV-IgM	□	□
● Hepatobiliary sonography / colour Doppler	□	□
● HCV: Anti-HCV, HCV-RNA	□	□
● HEV: Anti-HEV-IgM, anti-HEV-IgG, HEV-RNA	□	□
● Hepatobiliary sonography/colour Doppler sonography of liver vessels/ endosonography/CT/MRC	□	□
● Alcoholism (AST/ALT ≥ 2)	□	□
● Acute recent hypotension history (particularly if underlying heart disease)	□	□
Group II (5 causes)		
● Complications of underlying disease(s) such as sepsis, metastatic malignancy, autoimmune hepatitis, chronic hepatitis B or C, primary biliary cholangitis or sclerosing cholangitis, genetic liver diseases	□	□
● Infection suggested by PCR and titer change for		
● CMV (anti-CMV-IgM, anti-CMV-IgG)	□	□
● EBV (anti-EBV-IgM, anti-EBV-IgG)	□	□
● HSV (anti-HSV-IgM, anti-HSV-IgG)	□	□
● VZV (anti-VZV-IgM, anti-VZV-IgG)	□	□
Evaluation of groups I and II		
● All causes-groups I and II—reasonably ruled out	+2	□
● The 7 causes of group I ruled out	+1	□
● 6 or 5 causes of group I ruled out	0	□
● Less than 5 causes of group I ruled out	-2	□
● Alternative cause highly probable	-3	□
6. Previous hepatotoxicity of the drug/herb		
● Reaction labelled in the product characteristics	+2	□
● Reaction published but unlabelled	+1	□
● Reaction unknown	0	□
7. Response to unintentional reexposure		
● Doubling of ALT with the drug/herb alone, provided ALT below 5N before reexposure	+3	□
● Doubling of ALT with the drug(s)/herb(s) already given at the time of first reaction	+1	□
● Increase of ALT but less than N in the same conditions as for the first administration	−2	□
● Other situations	0	□
Total score for the case		□

**Table 3:** Updated RUCAM for the cholestatic or mixed liver injury of DILI and HILI.

Items for Cholestatic or Mixed Liver Injury	Score	Result
1. Time to onset from the beginning of the drug/herb		
● 5–90 days (rechallenge: 1–90 days)	+2	□
● <5 or >90 days (rechallenge: >90 days)	+1	□
Alternative: Time to onset from cessation of the drug/herb		
● (except for slowly metabolized chemicals: ≤30 days)	+1	□
2. Course of ALP after cessation of the drug/herb		
Percentage difference between ALP peak and N		
● Decrease ≥ 50% within 180 days	+2	□
● Decrease < 50% within 180 days	+1	□
● No information, persistence, increase, or continued drug/herb use	0	□
3. Risk factors		
● Alcohol use current drinks/d: >2 for women, >3 for men)	+1	□
● Alcohol use (current drinks/d: ≤2 for women, ≤3 for men)	0	□
● Pregnancy	+1	□
● Age ≥ 55 years	+1	□
● Age < 55 years	0	□
4. Concomitant use of drug(s)/herb(s)		
● None or no information	0	□
● Concomitant drug/herb with incompatible time to onset	0	□
● Concomitant drug/herb with compatible or suggestive time to onset	−1	□
● Concomitant drug/herb known as hepatotoxin and with compatible or suggestive time to onset	−2	□
● Concomitant drug/herb with evidence for its role in this case (positive rechallenge or validated test)	−3	□
5. Search for alternative causes	Tick if negative	Tick if not done
Group I (7 causes)		
● HAV: Anti-HAV-IgM	□	□
● HBV: HBsAg, anti-HBc-IgM, HBV-DNA	□	□
● HCV: Anti-HCV, HCV-RNA	□	□
● HEV: Anti-HEV-IgM, anti-HEV-IgG, HEV-RNA	□	□
● Hepatobiliary sonography/colour Doppler sonography of liver vessels/endosonography/CT/MRC	□	□
● Alcoholism (AST/ ALT ≥ 2)	□	□
● Acute recent hypotension history (particularly if underlying heart disease)	□	□
Group II (5 causes)		
● Complications of underlying disease(s) such as sepsis, metastatic malignancy, autoimmune hepatitis, chronic hepatitis B or C, primary biliary cholangitis or sclerosing cholangitis, genetic liver diseases	□	□
● Infection suggested by PCR and titer change for		
● CMV (anti-CMV-IgM, anti-CMV-IgG)	□	□
● EBV (anti-EBV-IgM, anti-EBV-IgG)	□	□
● HSV (anti-HSV-IgM, anti-HSV-IgG)	□	□
● VZV (anti-VZV-IgM, anti-VZV-IgG)	□	□
Evaluation of group I and II		
● All causes—groups I and II—reasonably ruled out	+2	□
● The 7 causes of group I ruled out	+1	□
● 6 or 5 causes of group I ruled out	0	□
● Less than 5 causes of group I ruled out	−2	□
● Alternative cause highly probable	−3	□
6. Previous hepatotoxicity of the drug/herb		
● Reaction labelled in the product characteristics	+2	□
● Reaction published but unlabelled	+1	□
● Reaction unknown	0	□
7. Response to unintentional reexposure		
● Doubling of ALP with the drug/herb alone, provided ALP below 2N before reexposure	+3	□
● Doubling of ALP with the drugs(s)/herbs(s) already given at the time of first reaction	+1	□
● Increase of ALP but less than N in the same conditions as for the first administration	−2	□
● Other situations	0	□
Total score for the case		□

### 3.2. Precursor Versions

Prior to the publication on the details of the original RUCAM in the two final reports in 1993 [8,9], preliminary details of precursor versions were published that resulted from consensus meetings [38,39,40] and were later partially incorporated in the final RUCAM version [8,9]. Published in 1988, the first pragmatic hepatotoxicity CAM based on the qualitative French CAM with chronological and clinical criteria was designed specifically for liver injury cases by considering some characteristic features [39] and formed a sophisticated basis for subsequent algorithms [8,9,40]. This early CAM version of 1988 [39] benefited from a prior version of 1987 [38] but still assessed the causality using qualitative criteria [39]. In 1990, substantial progress was made on a standard definition of DILI by differentiating between the hepatocellular, the cholestatic, and the mixed liver injury [40] that led in 1993 to the publication of the final and original RUCAM, which identified and scored quantitatively DILI specific key characteristics [8,9].

## 4. Original RUCAM

### 4.1. Consensus Meetings

Assessing the link between an adverse event and the drug by a formal causality assessment is still a challenging exercise despite the number of published methods [8]. All previous methods combine criteria and conclude on the strength of the link qualified as, for example, “compatible”, “suggestive” or “inconclusive”. These terms have never been accurately defined, leaving room for interpretation by the assessor with a major consequence on the results and then on the method itself, which will have a low reproducibility. In addition, the weight of each criterion was usually not adapted to the injured organ, decreasing further the specificity of the method. To face these issues, a broad consensus among experts on a new method for a drug causality assessment with focus on the liver was to be found. Experts were convened to organ-oriented international consensus meetings with the objectives to define adverse events and reach a consensus on criteria for causality assessment [38,39,40]. Preceding versions were extended, specified, and quantified, while additional criteria have been introduced and weights attributed [39,40]. This led to the publication of the original RUCAM as the novel CAM to be applied to suspected cases of DILI [8]. The reproducibility of RUCAM was tested by an independent team and the validity of this novel method was evaluated with reports including positive rechallenge as gold standard [9].

Briefly, to overcome experts’ and clinicians’ previous problems with organ-unrelated and unstructured evaluations lacking defined and scored items that commonly resulted in debated causality assignments, definitions of terms related to liver injuries and chronological criteria were developed in a consensus meeting by eight experts in hepatology from six countries. Among these experts were J. P. Benhamou (France), J. Bircher (Germany), G. Danan (France), W. C. Maddrey (USA), J. Neuberger (UK), F. Orlandi (Italy), N. Tygstrup (Denmark), and H. J. Zimmerman (USA) [8,9]. These experts in the field co-evaluated DILI cases for case characteristics, hepatotoxicity criteria, liver injury pattern, and reexposure criteria; they standardized DILI case assessment with specific and quantitative items; and they all received appropriate credits for their ambitious contribution and final approval of the original RUCAM criteria [8,9]. Later on, and partially based on the results of this consensus meeting, the original RUCAM was developed and validated by the team of Roussel Uclaf. RUCAM was initially developed for assessment of a single drug containing a single synthetic chemical but may well be used for a single herb or dietary supplement containing multiple chemical ingredients, but it does not allow causality attribution to one of the ingredients.

RUCAM considers all core elements of hepatotoxicity, is specific and well validated for hepatotoxicity, structured, itemized, scored, and quantitative. This facilitates transparent documentation of each scored RUCAM item and provides a total score for each patient with suspected DILI or HILI, whereby each used drug, herb, or dietary supplement needs a separate RUCAM assessment [8,9].

### 4.2. Case Definition

RUCAM was the first CAM worldwide that ever established valid criteria of a liver injury caused by a hepatotoxic drug reaction, using multiples of N (upper limit of normal) of liver tests (LTs) as diagnostic criterion or threshold [8,9], eliminating thereby cases with unspecific increased liver enzymes lacking clinical relevance.

### 4.3. Liver Injury Classification

RUCAM also was the first CAM that ever recognized the importance of various types of liver injury by drugs for a robust causality assessment [8,9]. Based on thorough DILI case analyses, three types of liver injury pattern emerged that showed striking differences of their clinical features and courses, with focus on challenge, dechallenge, and reexposure characteristics. These three types were classified as hepatocellular injury, cholestatic liver injury, and mixed liver injury. Due to the variability of their clinical features, specific key items and individual scores had to be defined for each of the three liver injury types. Subsequent analyses led to the conclusion that for the causality assessment, only two instead of three RUCAM versions are necessary, one for the hepatocellular injury and the other one for the cholestatic liver injury and the mixed liver injury with its predominant cholestatic features [8,9].

### 4.4. Elements with Individual Scoring

The original RUCAM comprises overall seven domains with liver related and hepatotoxicity specific core elements, is well structured, user-friendly, and clearly quantitative rather than vaguely qualitative as its scoring system includes all individual elements [8,9]. Core elements of the original RUCAM include: challenge features as time period from beginning until cessation of drug intake in relation to disease onset or from the cessation of drug use to the onset of the liver injury; dechallenge characteristics with course of LTs after cessation or continuation of the drug use; risk factors such as alcohol use, age and pregnancy; co-medication with other drugs or herbs; search for alternative causes; available information on previous drug hepatotoxicity; and response to unintentional reexposure, as intentional reexposures for diagnostic purposes are obsolete and unethical due to high risks associated with this test.

### 4.5. Sensitivity, Specificity, and Predictive Values

The original RUCAM [8] was validated by cases with known positive reexposure as external and gold standard [9]. Indeed it was recognized that expert’s opinion is too variable between observers and among a single observer to validate a causality assessment method but a positive reexposure test as a gold standard exists: the best but retrospective evidence that a compound was the cause of DILI or HILI was provided if the same type of liver injury was reproduced after an unintentional reexposure by the same compound at the same or even at a lower dose [44]. This type of data is difficult to find because it is harmful to intentionally reexpose the patient to a suspect drug. The risk is high to induce a more serious liver injury and sometimes a fatal outcome. Nevertheless, cases of DILI with unintentional reexposure by a suspect drug have been published, and these cases have been taken to validate the original RUCAM. The details of validation are described elsewhere [9]. Briefly, the principle was to assess with RUCAM each drug involved in 49 cases and to verify whether the score obtained (before reexposure) by the culprit drug was the highest one among those taken by the patient. Similarly and with another batch of 28 cases, RUCAM was applied to the drugs that were not the culprit ones and verify whether the score was the lowest one. The first step of the validation process was to check that the score including the criterion of positive rechallenge was discriminant enough to separate without overlapping the drugs that induced a liver injury and the others. This was quite clear since the final score was between –1 to +4 for the non-culprit drugs and +6 to +13 for the culprit drugs. Then, the second step was to evaluate the drugs without including the weight of reexposure and to determine the score range of the culprit and non-culprit drugs. Under these conditions, there was a score overlapping of the two drug categories in the zone of “possible” (score +3 to +5). This was expected since drugs administered at the same time for the same duration of treatment and with the same knowledge on their previous hepatotoxicity cannot be discriminated even for a clinician. Finally, RUCAM-based assessment has shown high sensitivity (86%), specificity (89%), positive predictive value (93%), and negative predictive value (78%) [9].

### 4.6. Shortcomings

Discussions with RUCAM users and experts of DILI and HILI as well as critical evaluations of the core items of the original RUCAM published in 1993 [8,9] suggested some adaptation of RUCAM to improve its accuracy and to minimize interobserver and intraobserver variability. Present improvements focus on recent new details of DILI and HILI characteristics that were not considered in the original RUCAM, as they were not available at the time of its publication [8,9]. In particular, special attention was now paid to few core elements that were ambiguous in its original domains such as alcohol use and exclusion of non-drug causes.

## 5. Updated RUCAM

The worldwide experience with the original RUCAM since 1993 [8,9] substantially facilitated the present actualization and modification of the original RUCAM, which now resulted in the publication of this current version, the updated RUCAM. Various points merit initial consideration (Figure 1 and Table 1) prior to starting with the updated RUCAM for causality assessment of suspected DILI and HILI cases (Table 2 and Table 3). Some general aspects (Figure 1) and specific operational information (Table 1) are essential and summarized to provide a correct use of the updated RUCAM with its subtypes for the acute hepatocellular liver injury (Table 2) and the acute cholestatic or mixed liver injury (Table 3). Finally, additional details are presented, which include a checklist of differential diagnoses of DILI and HILI (Table 4) and specific criteria for a positive result following an unintentional reexposure (Table 5).

**Table 4:** Checklist of differential diagnoses of DILI and HILI. This tabular listing, although not comprehensive, is to be used as a guide and in connection with the updated RUCAM (Table 2 and Table 3), derived from a previous publication [11]. Abbreviations: AAA, Anti-actin antibodies; AMA, Antimitochondrial antibodies; ANA, Antinuclear antibodies; ASGPR, Asialo-glycoprotein-receptor; BMI, Body mass index; CT, Computed tomography; CYP, Cytochrome P450; PDH, Pyruvate dehydrogenase; HAV, Hepatitis A virus; HBc, Hepatitis B core; HBV, Hepatitis B virus; HCV, Hepatitis C virus; HEV, Hepatitis E virus; HILI, Herb induced liver injury; HIV; human immunodeficiency virus; LKM, Liver kidney microsomes; LP, Liver-pancreas antigen; LSP, Liver specific protein; MRC, Magnetic resonance cholangiography; MRT, Magnetic resonance tomography; p-ANCA, Perinuclear antineutrophil cytoplasmic antibodies; PCR, Polymerase chain reaction; RUCAM, Roussel Uclaf Causality Assessment Method; SLA, Soluble liver antigen; SMA, Smooth muscle antibodies; TSH, Thyroid stimulating hormone; TTG, Tissue transglutaminase.

Differential Diagnosis	Diagnostic Parameters	Diagnostic Exclusion Done for Patient’s Assessment		
		Yes	No	Partial
● Hepatitis A virus (HAV)	Anti-HAV-IgM	□	□	□
● Hepatitis B virus (HBV)	HBV-DNA, anti-HBc-IgM	□	□	□
● Hepatitis C virus (HCV)	HCV-RNA, anti-HCV	□	□	□
● Hepatitis E virus (HEV)	HEV-RNA, titer change for anti-HEV-IgM/anti-HEV-IgG	□	□	□
● Cytomegalovirus (CMV)	CMV-PCR, titer change for anti-CMV-IgM/anti-CMV-IgG	□	□	□
● Epstein Barr virus (EBV)	EBV-PCR, titer change for anti-EBV-IgM/anti-EBV-IgG	□	□	□
● Herpes simplex virus (HSV)	HSV-PCR, titer change for anti-HSV-IgM/anti-HSV-IgG	□	□	□
● Varicella zoster virus (VZV)	VZV-PCR, titer change for anti-VZV-IgM/anti-VZV-IgG	□	□	□
● Other virus infections	Specific serology of Adenovirus, Coxsackie-B-Virus, Echovirus, Measles virus, Rubella virus, Flavivirus, Arenavirus, Filovirus, Parvovirus, HIV, and others	□	□	□
● Other infectious diseases	Specific assessment of bacteria, fungi, parasites, worms, and others	□	□	□
● Autoimmune hepatitis (AIH) type I	Gamma globulins, ANA, SMA, AAA, SLA/LP, Anti-LSP, Anti-ASGPR	□	□	□
● Autoimmune hepatitis (AIH) type II	Gamma globulins, Anti-LKM-1 (CYP 2D6), Anti-LKM-2 (CYP 2C9), Anti-LKM-3	□	□	□
● Primary biliary cholangitis (PBC)	AMA, Anti PDH-E2	□	□	□
● Primary sclerosing cholangitis (PSC)	p-ANCA, MRC	□	□	□
● Autoimmune cholangitis (AIC)	ANA, SMA	□	□	□
● Overlap syndromes	See AIH, PBC, PSC, and AIC	□	□	□
● Non alcoholic steatohepatitis (NASH)	BMI, insulin resistance, hepatomegaly, echogenicity of the liver	□	□	□
● Alcoholic liver disease (ALD)	Patient’s history, clinical and laboratory assessment, other alcoholic disease(s)	□	□	□
● Drug induced liver injury (DILI) or herb induced liver injury (HILI)	Patient’s history, clinical and laboratory assessment, sonography, use of the updated RUCAM	□	□	□
● Cocaine, ecstasy and other amphetamines	Toxin screening	□	□	□
● Rare intoxications	Toxin screening for household and occupational toxins	□	□	□
● Hereditary hemochromatosis	Serum ferritin, total iron-binding capacity, genotyping for C2824 and H63D mutation, hepatic iron content	□	□	□
● Wilson disease	Copper excretion (24 h urine), ceruloplasmin in serum, free copper in serum, Coombs-negative hemolytic anemia, hepatic copper content, Kayser-Fleischer-ring, neurologic-psychiatric work-up, genotyping	□	□	□
● Porphyria	Porphobilinogen in urine, total porphyrines in urine	□	□	□
● α_1_—Antitrypsin deficiency	α_1_—Antitrypsin in serum	□	□	□
● Biliary diseases	Clinical and laboratory assessment, hepatobiliary sonography, MRC	□	□	□
● Pancreatic diseases	Clinical and laboratory assessment, sonography, CT, MRT	□	□	□
● Celiac disease	TTG antibodies, endomysium antibodies, duodenal biopsy	□	□	□
● Anorexia nervosa	Clinical context	□	□	□
● Parenteral nutrition	Clinical context	□	□	□
● Cardiopulmonary diseases	Cardiopulmonary assessment of congestive heart disease, myocardial infarction, cardiomyopathy, cardiac valvular dysfunction, pulmonary embolism, pericardial diseases, arrhythmia, hemorrhagic shock, and various other conditions	□	□	□
● Addison’s disease	Plasma cortisol	□	□	□
● Thyroid diseases	TSH basal, T4, T3	□	□	□
● Grand mal seizures	Clinical context of epileptic seizure (duration > 30 min)	□	□	□
● Heat stroke	Shock, hyperthermia	□	□	□
● Polytrauma	Shock, liver injury	□	□	□
● Systemic diseases	Specific assessment of sarcoidosis, amyloidosis, metastatic tumor, sepsis, and others	□	□	□
● Other diseases	Clinical context	□	□	□

**Table 5:** Conditions of unintentional reexposure tests in suspected DILI and HILI cases.

Reexposure Test Result	Hepatocellular Injury		Cholestatic or Mixed Liver Injury	
	ALTb	ALTr	ALPb	ALPr
● Positive	<5N	≥2ALTb	<2N	≥2ALPb
● Negative	<5N	<2ALTb	<2N	<2ALPb
● Negative	≥5N	≥2ALTb	≥2N	≥2ALPb
● Negative	≥5N	<2ALTb	≥2N	<2ALPb
● Uninterpretable	<5N	n.a.	<2N	n.a.
● Uninterpretable	n.a.	≥2ALTb	n.a.	≥2ALPb
● Uninterpretable	n.a.	n.a.	n.a.	n.a.

Conditions and criteria for an unintentional reexposure test, adapted from a previous report [14]. Accordingly, required data for the hepatocellular type of liver injury are the ALT levels just before reexposure, designed as baseline ALT or ALTb, and the ALT levels during reexposure, designed as ALTr. Response to reexposure is positive, if both criteria are met: first, ALTb is below 5N with N as the upper limit of the normal value, and second ALTr ≥2ALTb. Other variations lead to negative or uninterpretable results. For the cholestatic (±hepatocellular) type of liver injury, corresponding values of ALP are to be used rather than of ALT. Abbreviations: ALP, Alkaline phosphatase; ALT, Alanine aminotransferase; n.a., not available.

### 5.1. Prospective Use

Conceptualized for prospective use, the updated RUCAM provides best results if applied prospectively rather than retrospectively (Table 1), ensuring completeness of case data sets and professional unbiased case evaluation. This avoids fruitless discussions among assessors around poor quality of case data and circumvents their problems of data interpretation with resulting interobserver and intraobserver variability. The individual items of the updated RUCAM are well defined and allow fast prospective collection of all diagnostic data while the patient is still under medical care. We have learnt that the original RUCAM often was applied in retrospect, although the philosophy of the original RUCAM favored its prospective use to collect in advance all data required for a valid causality evaluation. Out of these reasons, all items of the original RUCAM had been transparently provided for prospective use [8]. Neglect of prospective use inevitably may cause major problems regarding data interpretation among assessing physicians and experts when case data are vague or inconsistent.

### 5.2. Case Classification

Causality assessment by the updated RUCAM requires prior evaluation of liver injury criteria and its pattern in each suspected case (Figure 1). Respective criteria are readily assessable by initial measurement of ALT (alanine aminotransferase) and ALP (alkaline phosphatase).

#### 5.2.1. Liver Injury Criteria

Liver injury is defined by increased serum activities of ALT of at least 5N and/or of ALP of at least 2N (Figure 1), best assessed simultaneously on the day of first presentation. These thresholds will increase the specificity of the hepatotoxicity causality assessment, eliminates false positive cases, and substantiates hepatotoxicity causality at a high level of probability. They are also in line with a recent consensus on DILI [18]. However, when ALT is within the normal range, ALP increases should be paralleled by increased γ-glutamyltranspeptidase or better 5’ nucleosidase to rule out isolated increases of ALP activities due to bone or another origin rather than hepatobiliary disease (Table 1).

#### 5.2.2. Liver Injury Pattern

In accordance with the original RUCAM [8], the updated RUCAM takes into account divergent laboratory constellation of the liver injury and provides two different subscales (Figure 1): one for the hepatocellular type of injury (Table 2) and the other one for the cholestatic or mixed type of injury (Table 3). These types can be differentiated using the ratio R, calculated as the ALT/ ALP activity measured at the time liver injury is suspected, with both activities expressed as multiples of N (Figure 1). The liver injury is hepatocellular if ALT > 5N and ALP ≤ N, or if both ALT and ALP are elevated, R ≥ 5; the liver injury is cholestatic if ALP > 2N and ALT ≤ N, or if both ALT and ALP are elevated, R ≤ 2; the liver injury is mixed if ALT > 5N and ALP > N and 2 < R < 5 (Figure 1). This classification of liver injury pattern clearly assigns each DILI or HILI case to the updated RUCAM, either for the hepatocellular injury (Table 2) or the cholestatic and mixed liver injury (Table 3).

### 5.3. Core Items

As for the original RUCAM [8], the updated RUCAM with its two subscales provides in few domains various core items and scorings that are different for the hepatocellular injury (Table 2) as compared to the cholestatic and mixed liver injury (Table 3). Few core items of the updated RUCAM are now more specified and consider recent diagnostic developments (Table 2 and Table 3), thereby differing from the original RUCAM [8]. However, the individual scores of the updated RUCAM remained unchanged as in the original RUCAM [8]; consequently, for the updated RUCAM there is no need of a new validation as this has comprehensively been done already for the original RUCAM [9].

#### 5.3.1. Time to Onset from the Beginning of the Drug/Herb Administration

Challenge criteria are well scored and clearly defined with a time frame between beginning of the drug/herb use with day 0 as the first day of intake and the onset of increased liver enzymes or symptoms (Table 2 and Table 3). Termination of drug/herb use prior to the onset requires an alternative scoring and consideration should be paid to slowly metabolized chemicals with prolonged half-lives (Table 2 and Table 3).

#### 5.3.2. Course of ALT or ALP after Cessation of Drug/Herb

Precise dechallenge criteria with scores reflect the natural course of ALT and ALP after cessation of the suspect product and are cornerstones of the updated RUCAM to facilitate causality assessment (Table 2 and Table 3). Treatment during the dechallenge phase with drugs such as steroids or ursodesoxycholic acid may mask the natural course and allows only a score of 0 reflecting no information. Relevant future time points for repeated ALT determinations on days 8 and 30 after cessation of the suspect product ensures completeness of prospective data collection in cases with hepatocellular injury; lack of information refers to missing ALT results directly after cessation or/and in the further course (Table 2). For the cholestatic and mixed liver injury, dechallenge results of ALP at least on day 180 after cessation are of relevance for causality assessment (Table 3).

#### 5.3.3. Risk Factors

Risk factors of current alcohol use, age and pregnancy as assessed by the original RUCAM [8] are included in the updated RUCAM, with clarifying details of alcohol use (Table 2 and Table 3).

##### Alcohol Use

According to data obtained, analyzed, and validated during the creation of the original RUCAM [8,9], thresholds for current alcohol use are now further specified and given separately for women (2 drinks/day) and men (3 drinks/day) (Table 2 and Table 3), calculating 10 g ethanol for each drink.

##### Age

Age ≥ 55 years is again included as risk factor with its respective score (Table 2 and Table 3), based on the validated results obtained from analyzed cases [8,9],

##### Pregnancy

Pregnancy is a risk factor only for the cholestatic and mixed liver injury (Table 3) [8], not for the hepatocellular injury (Table 2).

#### 5.3.4. Concomitant Drug(s) and Herb(s)

Concomitant use of drugs and herbs is a crucial item that is best inquired and documented at first presentation when liver injury is suspected. Details of a temporal association and potential hepatotoxic features of the used product are to be assessed and documented (Table 2 and Table 3). For reasons of comparison and transparency, each comedicated drug or herb requires a separate analysis by the complete updated RUCAM. In patients with use of multiple drugs or herbs, the final causality should be attributed primarily to the product with the highest score achieved with the updated RUCAM (Table 2 and Table 3) [16,17].

#### 5.3.5. Search for Alternative Causes

In this domain, the updated RUCAM scale considers the clinically most relevant alternative causes and complications of underlying disease(s) (Table 2 and Table 3). Antibodies are important tools but require assessment and approval by regulatory agencies. Problems of diagnosing infections by hepatitis E virus (HEV) are evident in the US with anti-HEV antibody tests that are not FDA approved [17,30,45]. Most importantly, titer changes of antibodies in suspected hepatic viral infections are to be evaluated in the further clinical course to confirm or disprove an ongoing virus infection.

Rare alternative causes are included in a checklist of differential diagnoses (Table 4) [11] as a reminder for the clinician that many diagnoses exist as alternatives to DILI and HILI. These other diagnoses are to be considered, excluded or verified in the context of clinical data and importance, financial resources, and benefit for the patient. Establishing alternative causes contributes to the accuracy of the updated RUCAM and often provides clues to possible specific therapies.

#### 5.3.6. Previous Hepatotoxicity

Hepatotoxicity listed in the product information sheet (e.g., Summary of product characteristics in the EU or product information in the US) must be checked, although the terms used to express the liver injury may vary and usually do not refer to specific definitions. If it is mentioned, then the hepatotoxicity is considered as known for that compound. If this toxicity is not mentioned, a quick literature search in PubMed and the well documented NIH LiverTox website [46,47] are recommended to determine whether the product has already been involved in published DILI and HILI.

#### 5.3.7. Response to Unintentional Reexposure

A positive reexposure test result is a hallmark and gold standard in DILI cases [9] and recognized by respective scores (Table 2 and Table 3). To classify a reexposure test as positive, few criteria are required, as specified (Table 5) [14]. The respective criteria were based on the conclusions of International Consensus Meetings in 1988 [39] and 1990 [40], as reviewed previously [8,9] and recently [14,48,49]. For the hepatocellular type of injury, the defining criteria are ALT levels before reexposure (designated as baseline ALT or ALTb), and reexposure ALT levels (designated as ALTr) (Table 5). The reexposure test is positive if ALTb is <5N and ALTr is ≥2ALTb, negative if one or both criteria are not fulfilled, and uninterpretable if data are lacking for one or both criteria. For the cholestatic or the mixed liver injury, the assessment criteria and interpretation of results are similar, with ALT replaced by ALP (Table 5).

#### 5.3.8. Final Scores

Each item of the updated RUCAM receives an individual score, and the sum of the individual scores provides the final score for the case (Table 2 and Table 3). With +14 down to −9 points, there is a wide range of the final scores, leading to the following causality levels: ≤0 points, excluded causality; 1–2, unlikely; 3–5, possible; 6–8, probable; and ≥9, highly probable (Table 2 and Table 3).

## 6. Validity of Updated RUCAM

### 6.1. Valid Causality Assessment

The original RUCAM was tested for its validity with the use of DILI cases confirmed by positive rechallenge taken as the gold standard and DILI cases where there was strong evidence that drugs were not the culprit drugs [8,9]. As the updated RUCAM does not change fundamentally the structure and the weights of the original method there is no reason to consider a change in its validity. Presently, all relevant clinical diagnostic parameters are included in the updated RUCAM (Table 2 and Table 3) and in the checklist (Table 4). There is little evidence that liver histology adds substantially to the diagnosis of DILI or HILI, as these lack specific histopathological features since they mimic all primary hepatic and biliary diseases [50]. Therefore, liver histology is not part of the diagnostic program of the updated RUCAM (Table 2 and Table 3). However, it is possible to take into account the results of a liver biopsy examination to exclude an alternative cause but certainly not to confirm DILI or HILI.

### 6.2. Correct Diagnoses

Results based on the updated RUCAM (Table 2 and Table 3) are quickly available for trained physicians with substantial clinical experience who then have to decide whether DILI or HILI is the most like diagnosis in their patients or any other differential diagnoses (Table 4). Their decision is crucial, as DILI and HILI require discontinuation of the offending product whereas other diagnoses may require specific therapeutic modalities. The patient will immediately profit from the correct diagnosis achieved after using the updated RUCAM (Table 2 and Table 3) and the checklist (Table 4).

### 6.3. Missed Diagnoses

**Figure 2:**
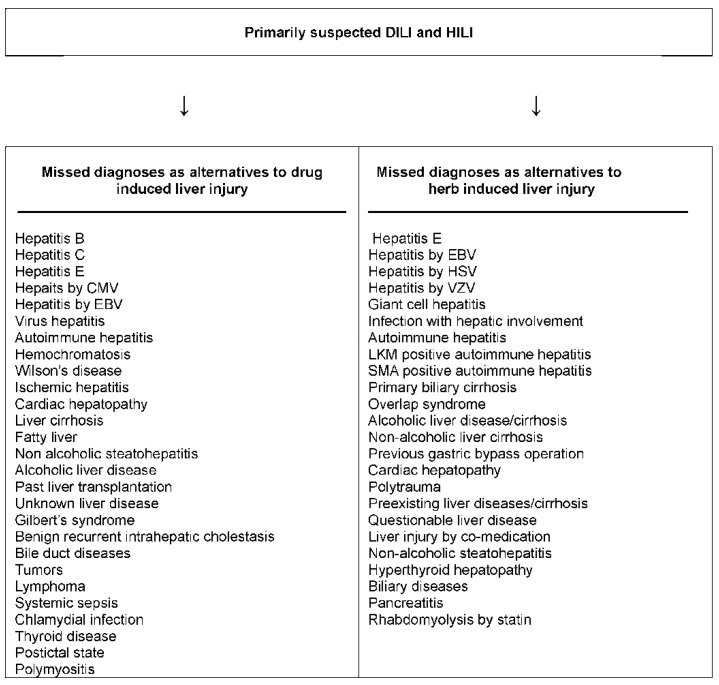
Missed diagnoses in cases of initially suspected hepatotoxicity by synthetic drugs or herbs. Adapted from previous reports [31,32], which provide the respective references for each missed diagnosis listed above. Abbreviations: CMV, Cytomegalovirus; EBV, Epstein Barr virus; HSV Herpes simplex virus; LKM, Liver kidney microsomes; SMA, Smooth muscle antibodies; VZV, Varicella zoster virus.

Unrecognized alternative causes to the liver injury are a real clinical problem when caring for patients with initially assumed but later not confirmed DILI [31] or HILI [32]; the long list of missed diagnoses in the setting of initially assumed injury cases is indeed threatening (Figure 2) [31,32]. More recent evidence suggests that the problem of missed diagnoses is multifaceted and caused by incomplete case data collection, poor case data analysis, problems of appropriate case data transfer from medical files to the manuscript, and unjustified upgrading of causality scores [16,17,51]. Missing the correct diagnosis may cause legal considerations that are better avoided by prior sophisticated clinical and regulatory approaches [14,15]. Effective treatments would have been available for some patients with missed diagnoses, a critical situation in the clinical context (Figure 2).

## 7. Updated RUCAM with Its Strengths and Challenges

### 7.1. Sophisticated Diagnostic Approach

Some complex diseases require a sophisticated diagnostic approach with preference of a valid scoring system composed of clearly defined diagnostic criteria. In this disease category belongs as examples not only the autoimmune hepatitis (AIH) that is well diagnosed by a specific score [52] and relapse while the suspected product has been discontinued, but also DILI and HILI that were validly diagnosed by the scoring causality method of the original RUCAM [8,9] and can now better be diagnosed with the updated RUCAM (Table 2 and Table 3). Instead of the well accepted original RUCAM [8,9] and its previous modifications [53], we now recommend the updated RUCAM (Table 2 and Table 3) for clinical, regulatory, publication, and expert purposes to validly establish causality in cases of suspected DILI and HILI.

### 7.2. Strengths

The updated RUCAM is a structured, liver and hepatotoxicity specific, quantitative method to assess causality of DILI and HILI cases, using specific and objective diagnostic elements with individual scores (Table 2 and Table 3). Derived from the well-validated original RUCAM [8,9], the updated RUCAM received substantial improvements and clarifications in some domains (Table 2 and Table 3) that will now allow better agreement among assessors and hence reducing interobserver and intraobserver variability. By providing accurately defined core elements, the aim was also to simplify the handling of the items without the need of external experts for the vast majority of cases. Its results can be adapted further to diagnostic and therapeutic measures and allows early planning and collection of all other relevant data to ensure completeness of case data sets. Assessment is achievable without involvement of external experts who are not commonly available in place and time when needed. Due to its user-friendly properties, the updated RUCAM can be viewed as a guide for the investigation of suspected DILI and HILI cases and will encourage both mandatory prospective evaluation and transparent data presentation to allow reassessment by other clinicians and scientists. The final scores provide reproducible causality gradings of highly probable down to excluded (Table 2 and Table 3). As an easily applicable scoring system, the updated RUCAM is a helpful bedside tool for physicians in care of patients with suspected DILI or HILI, starting with the assessment at their first presentation

### 7.3. Mandatory Systematic Documentation for Data Transparency

Each evaluation of a DILI or HILI case by the updated RUCAM requires mandatory documentation through assessing physician, hospital, regulatory agency, and pharmaceutical company. For reasons of transparency, each item with the achieved individual and final score is to be provided, using the published documents of the updated RUCAM (Table 2 and Table 3). Published case reports or case series also should provide appropriate documentation including a list of available or lacking case data that were analyzed. Examples how to manage this systematic data documentation even for a large number of cases are available elsewhere [15]. For journals with space restriction, supplementary files will circumvent this limitation, considering that lack of a complete and systematic RUCAM-based documentation will invalidate the results.

Transparency is a hallmark of RUCAM with its clear core elements and individual scorings (Table 2 and Table 3) that are easily applicable to any DILI or HILI report destined for regulatory agencies and pharmaceutical companies or publication [15]. In this context, special care is required to publish the result of each core element with its score for each patient and separately for each product taken at the same time. Unfortunately, many reports lack transparent presentation of required RUCAM details, making these reports difficult to assess. In fact, this approach of the authors to provide incomplete case and RUCAM data raises issues to scientists, physicians and regulators aiming to reassess the published cases. In a worst case scenario, only the final core is published. It is hoped that editors will require such transparency before publishing DILI and HILI case reports or case series lacking these details of causality assessment (Table 2 and Table 3).

### 7.4. Robust Framework by Sequential Assessment

Clearly, physicians need a robust framework to establish the diagnosis of DILI and HILI in the early clinical course when the diagnosis is suspected and the disease is unfolding, and not thereafter and retrospectively when the disease has vanished. Therefore, a pragmatic stepwise approach is recommended. As the first step, a careful clinical assessment is necessary, best summarized as a case narrative [15] and combined with the use of the updated RUCAM (Table 2 and Table 3) and the checklist of differential diagnoses (Table 4). In the second step and if uncertainty remains, an optional expert opinion or regulatory evaluation may follow, using as basic common tool the scored items of the updated RUCAM established before; for this second step, a local expert panel can be involved in any country, in the United States the Drug-Induced Liver Injury Network (DILIN) might be operative [3].

### 7.5. Open for Worldwide Use

The updated RUCAM with its basic and objective core items and the associated scores (Table 2 and Table 3) is well prepared for international use, as was the original RUCAM successfully by pharmaceutical companies, multiple international registries and regulatory agencies from European, Asian, and South American countries [14]. Therefore, an internationally harmonized, uniform approach of causality assessment is preferred, using the case narrative [15], the updated RUCAM scale (Table 2 and Table 3) and the checklist for differential diagnoses of DILI and HILI (Table 4) as basic tool, optionally an expert panel reassessing the narrative of clinical case characteristics and the quantified RUCAM items obtained by the treating physician. This stepwise approach will facilitate completeness of case data and ensure case data transparency and comparability. It is also a chance for an internationally harmonized approach of causality assessment and improves the acceptance of published case reports or case series on DILI and HILI. It would be helpful if uniformity of DILI and HILI criteria including specific scoring is established worldwide so published data across countries and their registries can be harmonized and easily interpreted across populations. Therefore, the updated RUCAM should best be considered as a standardized approach for causality assessment of DILI and HILI cases, both for the attending physician and all later stages by experts if needed. Using a single assessment method allows valid and reproducible comparisons of different assessment outcomes.

### 7.6. Limitations

The updated RUCAM is as good as physicians and assessors are handling this method and consider basic liver tests for the liver injury classification (Figure 1), the specific operational information (Table 1), details of core elements and their scoring (Table 2 and Table 3), differential diagnoses (Table 4), and the reexposure criteria (Table 5). RUCAM has not been designed for chronic DILI and HILI or when a suspected injury occurs on pre-existing liver disease, both complex conditions where an expert panel of hepatologists would provide a more accurate approach especially for the timing of the events and the exclusion of alternative causes.

**Table 6:** Core elements of the updated RUCAM as compared to other causality assessment methods. Data for RUCAM are derived from the updated RUCAM (Table 2 and Table 3 and Figure 1), for MV from the report of Maria and Victorino [41], For DILIN from the Drug Induced Liver Injury Network method [33,54,55], for Naranjo from the report of Naranjo *et al.* [56], for the WHO from the WHO database [57], and for the ad-hoc approach from Kaplowitz [58]. The symbol + shows that this specific item is published, and the symbol 0 indicates lacking publication. Abbreviations: ALT: Alanine aminotransferase; ALP: Alkaline phosphatase; CMV: Cytomegalovirus; EBV: Epstein Barr virus; HAV: Hepatitis A virus; HBV: Hepatitis B virus; HCV: Hepatitis C virus; HEV: Hepatitis E virus; HSV: Herpes simplex virus; VZV: Varicella zoster virus

Items	RUCAM	MV	DILIN	Naranjo	WHO	*Ad Hoc*
● Time frame of latency period (score)	+	+	0	0	0	0
● Time frame of dechallenge (score)	+	+	0	0	0	0
● Recurrent ALT or ALP increase (score)	+	0	0	0	0	0
● Definition of risk factors (score)	+	0	0	0	0	0
● All comedications (score)	+	0	0	+	0	0
● Individual comedication (score)	+	0		0	0	0
● Search for individual alternative causes (score)	+	+	0	0	0	0
● Verified exclusion of specific alternative causes (score)	+	+	0	0	0	0
● All specifically assessed HAV, HBV, HCV, HEV (score)	+	0	0	0	0	0
● All specifically assessed CMV, EBV, HSV, VZV (score)	+	0	0	0	0	0
● Evaluation of cardiac hepatopathy (score)	+	+	0	0	0	0
● Liver and biliary tract imaging (score)	+	0	0	0	0	0
● Color Doppler sonography of liver vessels (score)	+	0	0	0	0	0
● Prior known hepatotoxicity (score)	+	+	0	+	0	0
● Search for unintended reexposure (score)	+	+	0	+	0	0
● Definition of unintended reexposure (score)	+	0	0	0	0	0
● Qualified criteria of unintended reexposure (score)	+	0	0	0	0	0
● Laboratory hepatotoxicity criteria	+	+	+	0	0	0
● Laboratory hepatotoxicity pattern	+	+	+	0	0	0
● Hepatotoxicity specific method	+	+	+	0	0	0
● Structured, liver related method	+	+	0	0	0	0
● Quantitative, liver related method	+	+	0	0	0	0
● Validated method (gold standard)	+	0	0	0	0	0

## 8. RUCAM and the Other Causality Assessment Methods

Due to its liver specificity and quantitative evaluation, the updated RUCAM should preferably be the method of choice to assess the product(s) that cause a liver injury (Table 2 and Table 3); nevertheless, a variety of other approaches attempted to assess the cause(s) of hepatic adverse reactions [33,34,53]. Only few of the other approaches were liver oriented, as most had been established to evaluate types of adverse reactions other than hepatotoxic ones. For reasons of comparison, some CAMs are listed as examples (Table 6). Overall, liver specific assessment methods such as the original RUCAM and the updated RUCAM are to be dissociated from liver unspecific ones.

### 8.1. Liver Specific Methods

Based on some principles of the original RUCAM as the first liver specific method [8,9], three other liver specific methods were developed including the scale of Maria and Victorino (MV) [41], the TKK scale named after the first three authors Takikawa, Takamori, Kumagi *et al.* [42], and the DILIN method of the DILIN group [33,54]. For various reasons, each of these three methods is still limited to the use of their authors due to major shortcomings [33,34,41,42,43,54].

#### 8.1.1. MV Scale

In an attempt to improve the original RUCAM [8], the MV scale was developed by deleting laboratory items and adding clinical elements, along with simplifying and changing the relative weight of elements in their algorithm [41], as discussed in detail [33,34,53]. As a shortened and modified version of the original RUCAM [8], the MV scale has fewer specific criteria; evaluates dechallenge as the time necessary for ALT or ALP to fall below 2N; and considers a shorter latency period [41]. It also asks for less accurate exclusion criteria of alternative causes; ignores concomitant drug or herb use; emphasizes drugs with more than five years marketing without published hepatotoxicity and overestimates extrahepatic manifestations [41]. Despite these major modifications, the performance indicators (specificity and sensitivity) including predictive values, and validation using a gold standard are not available for the MV scale [41]. Compared to the updated RUCAM, the MV scale shows major differences (Table 6). Considering also critical comments on shortcomings [33,34,53], the MV scale is not commonly recommended for assessing causality in suspected DILI and HILI cases and is certainly not a substitute for the RUCAM.

#### 8.1.2. TTK Scale

The TTK scale was established for DILI cases specifically in Japan [42,43] and is another attempt to modify the original RUCAM [8] with different evaluations of the chronology, exclusion of comedication, inclusion of the drug lymphocyte stimulation test (DLST) and eosinophilia in their assessment [42,43]. The TTK scale is widely used in Japan [42], as recently reviewed [43]. Limited access and lack of standardization have prevented general clinical use of the DLST and consequently TTK scale applications outside Japan [34,36]; this may be due to methodological difficulties with false positive and false negative DLST [36,43]. For clinicians, the TTK scale cannot replace the original RUCAM [36].

#### 8.1.3. DILIN Method

Members of the DILIN group provided their assessment method [33,54] that is liver specific since it used many core items of the original RUCAM [8] However, it ignores missing important items [55] and lacks a system that scores key elements, as does the original RUCAM [8] or the updated RUCAM (Table 2, Table 3 and Table 6). Since individual weighing and scoring of key items are lacking and undiscussed [33,54], results published by the DILIN group using their method are not transparent [3,6,13,30,54] and not available for reassessment [53]. This lack of scoring is one of the most disturbing shortcomings of this method as discussed in detail earlier [53]. With limited use by other authors in the DILI literature, the DILIN method requires an expert panel on expert’s opinion [33], in contrast to the original RUCAM [8,9] and the updated RUCAM (Table 2 and Table 3). Consequently, the DILIN method is not available to physicians in need of early results for therapeutic decisions and in no way an appropriate substitute for RUCAM [53]. The DILIN method was not validated by any established gold standard [33] as the original RUCAM [8,9].

### 8.2. Liver Unspecific Methods

As opposed to the liver specific core elements of the original RUCAM [8,9] and the updated RUCAM (Table 2 and Table 3), such elements are not part of other methods that were established to assess causality in patients with all kinds of adverse events but not specifically those occurring in the liver [53]. Among these liver unspecific methods is the Naranjo scale [56], the WHO global introspection method, WHO method in short [57], the *ad hoc* approach [58], and the KL method named after Karch and Lasagna [59]. Surprisingly, all four methods were applied in suspected DILI or HILI cases, with preference of the first three [53], which therefore were compared with the liver specific methods (Table 6). Regulatory agencies appear to prefer these three unspecific methods in liver injury cases [56,57,58] since this ensures high case numbers attributed to drugs or herbs despite low data quality, as discussed in detail for the Naranjo scale [4,60,61,62,63,64,65], the WHO method [66,67,68,69,70,71], and the *ad hoc* approach [66,72,73], and as to be discussed for the recent *ad hoc* approach [74]. All liver unspecific methods are obsolete for causality assessment of DILI and HILI cases [53]. Furthermore, highly questionable are assessments based on MedWatch cases [60,74,75] with their known poor data quality [60,75] as reported in the majority by non-professionals [74]. Therefore, poorly documented cases and use of inappropriate causality assessment methods constitute a hazardous combination certainly not meeting the requirements of a solid regulatory work.

#### 8.2.1. Naranjo Scale

The use of the liver unspecific Naranjo scale [56] in suspected DILI and HILI cases is problematic [4,53,60,61,62,63,64,65,76] as criteria of hepatotoxicity and reexposure conditions, specific time to onset, criteria for recovery time, and critical diagnoses to exclude are not even unconsidered (Table 6) [53]. It also relates toxic drug reactions to general pharmacological drug actions rather than to idiosyncratic reactions like rare DILI or HILI. The items include drug concentrations and monitoring, dose relationship including decreasing dose, placebo response, and cross-reactivity, using unidentified objective evidence. Since these items are irrelevant for DILI and HILI, they have less sensitivity for rare and idiosyncratic reactions prevalent in liver injury [53,56]. In addition, this scale results in a possible causality even in the absence of essential data, by virtue of the patient simply took the suspect agent [4,53]. Problems related to the Naranjo scale were also not resolved [53] when the United States Pharmacopeia (USP) used its own modified, shortened, and not validated Naranjo version with only five instead of the original ten items [64]. Lacking test validity and reproducibility [76], the use of this method has raised concern about judgment validity by the USP [4,61]. In essence, the use of the Naranjo scale for suspected DILI and HILI should not be recommended anymore.

#### 8.2.2. WHO Method

The WHO method was developed for general adverse events and is not liver specific, was not developed or validated for DILI or HILI cases [57], and does not consider hepatotoxicity related characteristics (Table 6). These shortcomings have raised major concern and led to the conclusion that this scale is neither appropriate for causality assessment in suspected hepatotoxicity cases nor has it advantages over other causality algorithms [53]. The WHO method is heavily disputed [53,66,67,68,69,70,71] and was not specifically mentioned, addressed, or discussed as causality assessment method for hepatotoxicity cases in relevant reports [53] including a recent statement of the NIH LiverTox [33]. This method is obsolete for hepatotoxicity case assessment.

#### 8.2.3. *Ad Hoc* Approach

No specific causality assessment method was used in two thirds of published DILI cases [77], implying that some kind of a clinical *ad hoc* approach tried to classify the causality of a case with its known shortcomings [53,58,66] including lack of specific and validated hepatotoxicity criteria and missing a scoring system (Table 6). When using this *ad hoc* approach, the physician may note the coincidence of a herbal or chemical drug use and will estimate the likelihood of a hepatotoxic reaction [58]. Results of these prima vista evaluations are fragile, disputed, not transparent, and not reassessable, as shown for the assessments by the Germany regulatory agency BfArM (Bundesinstitut für Arzneimittel und Medizinprodukte, Federal Institute for Drugs and Medicinal Products) [66,72,73] and the FDA in the USA [74]. Although disputed [66] in connection with the German BfArM [72,73], FDA regulators also applied this dubious *ad hoc* approach retrospectively in MedWatch cases [74], known for their poor data quality [60,75] as reported mostly by non-professionals commonly not familiar case details and aware of the specific issues [74]. The most reliable assessment would have been to provide transparent data and to include RUCAM with focus on few well documented cases, reducing the risk of overreporting and providing scientific quality rather than likely unjustified case quantity. Reports based on the *ad hoc* approach are rather disappointing and the method should not be applied nor recommended for suspected DILI and HILI causality assessment.

## 9. RUCAM and Its International Use

There is general agreement that RUCAM is the most commonly used causality assessment method to identify suspected DILI and HILI cases [33,77,78]. Its worldwide use is well documented in the literature, considering reports by international registries, regulatory agencies and their associated groups (Table 7) [1,2,5,76,79,80,81,82,83,84,85,86,87,88,89,90,91,92,93,94,95] and the large number of reports on individual cases and case series (Table 8) [5,8,9,16,17,60,70,73,96,97,98,99,100,101,102,103,104,105,106,107,108,109,110,111,112,113,114,115,116,117,118,119,120,121,122,123,124,125,126,127,128,129,130,131,132,133,134,135,136,137,138,139,140,141,142,143,144,145,146,147,148,149,150,151,152,153,154,155,156,157,158,159,160,161,162,163,164,165,166,167,168,169,170,171,172,173]. RUCAM has been used to identify DILI and HILI events in case studies of prescription drugs [8,9,112,172], herbal medications [14,168], regulatory evaluations [81,86,87,88,90,92,94], epidemiological studies [2,99,113,173], genotyping studies [83,100], phase I clinical studies [154] and long-term post marketing clinical trials [5,102,158], just to name a few examples.

**Table 7:** Listing of selected international registries and regulatory agencies, and associated groups that applied RUCAM in suspected DILI and HILI cases.

Cases	Suspected Products	Country or Region	Group/Agency	Year	First Author
DILI	Multiple synthetic drugs	Spain Europe	Spanish Group for the Study of the Drug-Induced Liver Disease, Malaga	2005	Andrade [1]
DILI	Multiple synthetic drugs	Spain Europe	Spain Hepatotoxicity Registry, Grupo de Estudio Para las Hepatopatías Asociadas a Medicamentos, Malaga	2006	Andrade [2]
HILI, DILI	Various herbal TCM, synthetic drugs	Singapore Asia	National University of Singapore	2006	Wai [79]
HILI	Lu Cha	Sweden Europe	Swedish Adverse Drug Reactions Advisory Committee	2007	Björnsson [80]
HILI	Black cohosh	Various countries Europe	European Medicines Agency	2007	EMA [81]
HILI	Herbs	Spain Europe	Spanish Liver Toxicity Registry	2008	García-Cortés [82]
DILI HILI	Multiple synthetic drugs, few herbs	Spain Europe	Spanish Group for the Study of Drug-induced Liver Disease	2008	García-Cortés [76]
DILI	Flucloxacillin	UK, other countries	DILIGEN Study & International SAE Consortium	2009	Daly [83]
DILI	Synthetic drugs	Serbia Europe	Medicines and Medical Devices Agency of Serbia, Belgrade	2010	Miljkovic [84]
HILI	*Polygonum multiflorum*	Korea Asia	Gyeongsang National University School of Medicine, Jinju/Sungkyunkwan University School of Medicine, Changwon	2011	Jung [85]
HILI	Various herbal TCM	Hong Kong	Hong Kong Herb-Induced Liver Injury Network (HK-HILIN), Hong Kong	2011	Chau [86]
DILI	Multiple synthetic drugs	Spain, other countries	Spanish DILI Registry, EUDRAGENE, DILIN, DILIGEN, and International SAEC.	2011	Lucena [87]
DILI	Synthetic drugs	Serbia Europe	Medicines and Medical Devices Agency of Serbia, Belgrade	2011	Miljkovic [88]
DILI	Statins	Iceland/Sweden Europe	National University Hospital Reykjavik/ University of Gothenburg/Swedish Adverse Drug Reactions Advisory Committee (SADRAC)	2012	Björnsson [5]
DILI	Various synthetic drugs (expected)	Spain Latin America	Spanish-Latin American Network on drug induced liver Injury, in progress	2012	Bessone [89]
DILI	Flupirtine	Germany Europe	Drug Commission of the German Medical Association	2012	Stammschulte [90]
HILI	Some Herbalife® products	USA, other countries	Various registries and groups	2013	Halegoua de Marzio [91]
DILI	Flupirtine	Germany Europe	Berlin Case-control Surveillance Study, German drug reaction reporting database	2014	Douros [92]
DILI	Anabolic and androgenic steroids	Spain, Latin America	Spanish DILI Registry and Spanish-Latin-American DILI Network	2015	Robles-Diaz [93]
DILI	Multiple synthetic drugs	Germany Europe	Berlin Case-control Surveillance Study	2015	Douros [94]
HILI DILI	Multiple dietary supplements and synthetic drugs	USA	Hawaii Department of Health	2015	Johnston [95]

**Table 8:** Listing of selected individual reports using RUCAM in suspected DILI and HILI cases.

Cases	Products	Country/Region	Year	First Author
DILI	Various synthetic drugs	France Europe	1993	Danan [8]
DILI	Various synthetic drugs	France Europe	1993	Bénichou [9]
DILI	Ketoprofen	France Europe	1998	Flamenbaum 96]
DILI	NSAIDs	Europe Europe	2003	Lucena [97]
HILI	Kava	Germany Europe	2003	Stickel [98]
DILI	Various synthetic drugs	Japan Asia	2003	Masumotuo [99]
DILI	Multiple synthetic drugs	Spain Europe	2004	Andrade [100]
DILI	Pioglitazone	France Europe	2004	Arotcarena [101]
DILI	Ximelagatran	USA, France, Sweden	2005	Lee W [102]
HILI	Ji Xue Cao	Argentina South America	2005	Jorge [103]
HILI	Lu Cha	France Europe	2005	Gloro [104]
DILI	Amoxicillin, Amoxicillin/Clavulanate	USA	2005	Fontana [105]
DILI	Various synthetic drugs	Sweden Europe	2006	De Valle [106]
HILI	Bo He, Chuan Lian Zi, and various other herbal TCM	Korea Asia	2006	Yuen [107]
HILI	Lu Cha	Spain Europe	2006	Jimenez-Saenz [108]
HILI	*Polygonum multiflorum*	Columbia South America	2006	Cárdenas [109]
DILI	Rofecoxib	Canada North America	2006	Yan [110]
DILI	Antibiotics	UK Europe	2007	Hussaini [111]
DILI	Atomoxetine	USA	2007	Stojanovski [112]
DILI	Various synthetic drugs	Sweden Europe	2007	Björnsson [113]
DILI	Flavoxate	Italy Europe	2007	Rigato[114]
HILI	Kava	Germany Europe	2008	Teschke [115]
HILI	Bai Xian Pi, Kudzu, Lu Cha, Yin Chen Hao	Korea Asia	2008	Kang [116]
HILI	Bai Xian Pi, Ci Wu Jia, Shou Wu Pian, Yin Chen Hao	Korea Asia	2008	Sohn [117]
DILI	Albedazole	Korea Asia	2008	Choi [118]
HILI	Indian Ayurvedic herbs	Germany Europe	2009	Teschke [119]
HILI	Green tea (*Camellia sinensis*)	Italy Europe	2009	Mazzanti [120]
HILI	Herbalife	Switzerland Europe	2009	Stickel [121]
HILI	*Corydalis speciosa*	Korea Asia	2009	Kang [122]
DILI	Black cohosh	Germany Europe	2009	Teschke [123]
HILI	Black cohosh	Germany Europe	2009	Teschke {124]
HILI	Ge Gen	Korea Asia	2009	Kim [125]
DILI	Montelukast	India Asia	2009	Harugeri [126]
DILI	Nimesulide	Italy Europe	2010	Licata [127]
DILI	Tadalafil	Morocco Africa	2010	Essaid [128]
HILI	Herbalife	Iceland Europe	2010	Jóhannsson [129]
HILI	H Shou Wu	Korea Asia	2010	Bae [130]
DILI	Antimicrobial agents	Thailand Asia	2010	Treeprasertsuk [131]
HILI	Aloe	Korea Asia	2010	Yang [132]
DILI	Cephalexin	USA	2010	Singla [133]
HILI	Kava	Germany Europe	2010	Teschke [134]
HILI	*Gynura segetum*	Hong Kong Asia	2011	Lin [135]
DILI	*Amiodarone*	Israel Europe	2011	Gluck[136]
DILI	Paracetamol	Spain Europe	2011	Sabaté [137]
HILI	Greater Celandine	Germany Europe	2011	Teschke [73]
HILI	Black cohosh	Germany Europe	2011	Teschke [60]
HILI	*Pelargonium sidoides*	Germany Europe	2012	Teschke [70]
DILI	Statins	Sweden Europe	2012	Björnsson [5]
DILI	Various dietary supplements	Iran Asia	2012	Timcheh-Hariri [138]
HILI	Greater Celandine	Germany Europe	2012	Teschke [139]
DILI	Etifoxine	France Europe	2012	Moch [140]
HILI	Juguju	Korea Asia	2012	Kim [141]
HILI	*Gynura segetum*	Hong Kong Asia	2012	Gao [142]
DILI	Varenicline	USA	2012	Sprague [143]
DILI HILI	Multiple synthetic drugs and herbs	KoreaAsia	2012	Suk [144]
DILI	Multiple synthetic drugs	China Asia	2012	Hou [145]
DILI	Etravirine	USA	2012	Nabha [146]
HILI	*Pelargonium sidoides*	Germany Europe	2012	Teschke [71]
DILI	Crizotinib	France Europe	2013	Ripault [147]
DILI	Methylprednisolone	France Europe	2013	Carrier [148]
DILI	Albendazole	Colombia South America	2013	Ríos [149]
HILI	Herbalife	Germany Europe	2013	Teschke [59]
DILI	Ibandronate	Belgium Europe	2013	Goossens [150]
DILI	Bosentan	USA	2013	Markova [151]
DILI	Cyproterone acetate	Italy Europe	2013	Abenavoli [152]
DILI	Various synthetic drugs	Iceland Europe	2013	Björnsson [153]
DILI	NSAID (investigational)	USA	2013	Marumoto [154]
HILI	Black cohosh	USA	2014	Adnan [155]
DILI	Volatile anesthetics	Australia	2014	Lin [156]
DILI	Multiple synthetic drugs	USA	2014	Cheetham [157]
DILI	Rivaroxaban	Switzerland Europe	2014	Russmann [158]
DILI	Daptomycin	USA	2014	Bohm [159]
DILI	Anastrazole	UK Europe	2014	Saiful-Islam [160]
HILI	Greater Celandine	Korea Asia	2014	Im [161]
DILI	Various synthetic drugs	USA	2014	Lim [162]
DILI HILI	Multiple synthetic drugs and herbal TCM	China Asia	2014	Hao [163]
DILI	Pomalidomide	USA	2014	Veluswamy [164]
DILI	Amoxicillin	USA	2014	Lin [165]
HILI	Various dietary supplements	USA	2014	Roytman [51]
DILI	Sofosbuvir	UK Europe	2015	Dyson J [166]
DILI HILI	Multiple synthetic drugs and dietary supplements	Germany Europe	2015	Teschke [16]
HILI	Lesser Celandine	Turkey Europe	2015	Yilmaz [167]
HILI	Green tea (Camellia sinensis)	Italy Europe	2015	Mazzanti [168]
DILI	Ipimimumab	Australia	2015	Tauquer [169]
DILI	Meloxicam	Korea Asia	2015	Son [170]
DILI	Rivaroxaban	USA	2015	Baig [171]
DILI	Bupropion, doxycycline	USA	2015	Tang [172]
DILI HILI	Multiple synthetic drugs and dietary supplements	Germany Europe	2016	Teschke [17]
DILI HILI	Multiple synthetic drugs and herbs	Korea Asia	2016	Woo [173]

Abbreviation; TCM, Traditional Chinese Medicine.

### 9.1. International Registries and Regulatory Agencies

The listing of selected international registries, regulatory agencies, and associated groups shows that RUCAM can be applied in suspected DILI and HILI cases in the regulatory context (Table 7). Presumably, other registries and regulatory agencies also use RUCAM but do not publish their approach. Considering only the published reports, European countries look predominate as roughly compared with other countries (Table 7). However, the use of the updated RUCAM in other countries should be encouraged to compare the risk of hepatotoxicity with the same approach. Among other regulatory agencies, EMA provided a good example how to apply RUCAM for regulatory purposes [81], and in Germany the switch from the debated WHO method or the *ad hoc* approach to RUCAM appears promising [90,92,94].

In the USA, the USP and the FDA look more cautious with RUCAM [64,74] while their preferences are the debated unspecific Naranjo scale [64] or the heavily disputed *ad hoc* approach [74]. However, a recent study of the Hawaii Department of Health applied RUCAM in a heterogeneous group of patients after use of multiple dietary supplements and synthetic drugs. They reported in two thirds of the cases the level “possible” of causality. Confounders included alcohol use (50%), up to five comorbidities (50%), and comedication with other dietary supplements (50%) and synthetic potentially hepatotoxic drugs such as Tylenol (acetaminophen) or NSAIDs (71%) [74]. What is also problematic in this report is the vague and broad inclusion criterion stating that the last exposure had to be within 60 days prior illness onset, too long and unacceptable time frame leading to a questionable temporal association [74]. As Hawaii lacks FDA-approved HEV antibody tests [30,45], HEV infection was not validly excluded in the reported Hawaii cases [16,17,51,74,154]. As shown in these examples, RUCAM can help to delineate case data quality and clarify that major uncertainties remain for causality assessment.

### 9.2. Published Reports

From 1993 a large number of individual cases or case series reports applied RUCAM, as it is shown in the listing of selected reports (Table 8). In most cases originated from many countries, reports described a “probable” or “highly probable” level of causality for a specific synthetic drug, herb, or dietary supplement based on RUCAM criteria. On the opposite, following case re-assessment utilizing RUCAM, causality had to be denied for some products such as herbs [16,17,59,60,71,73,134,139] and dietary supplements [16,17]. Similarly, RUCAM-based assessment revealed that initially suspected amiodarone-induced liver injuries were likely due to ischemic hepatitis consecutive to severe arrhythmia or acute cardiac failure [136].

## 10. Perspectives

With the updated RUCAM (Table 2 and Table 3), a major step forward has been made to facilitate causality assessment in suspected DILI and HILI cases and should replace previous and unspecific methods. Its prospective use by clinicians is strongly recommended to ensure collection of complete data and avoid retrospective discussions on data quality. Although RUCAM was several times challenged on its apparent complexity, clinicians in general and hepatologists in particular, are quite satisfied with the logical and complete approach of the suspected DILI and HILI as guided by the method and mentioned by the users in the papers. In addition, it is rarely necessary to train people on the use of RUCAM as the method follows the natural and clinical reasoning at the patient bedside. Clear causality attribution in suspected DILI and HILI cases is essential to provide the appropriate therapy and to protect consumers from health hazards. This is the main reason why RUCAM should be incorporated into the list of the scientific methods to be used in the benefit-risk assessment approach when hepatotoxicity is one of the identified risks of a product. Whether in drug development or in post marketing, the case-by-case analysis is critical in the decision-making approach. All the medical reviewers, either from the regulatory or the company side, will be able to check the case assessment using RUCAM hence increasing confidence on the risk evaluation and therefore on the risk management. Indeed, having a series of relevant and correctly assessed cases with RUCAM allows for a better definition of the risk characteristics and decision to take appropriate measures to minimize this risk.

## 11. Conclusions

The major strength of the updated RUCAM is its potential as a standard scale for DILI and HILI to assess causality by attending physicians, regulatory agencies, expert panels, and the scientific community. It provides a straightforward application in liver and hepatotoxicity specific domains with scored items. This allows data comparability and transparency, reassessment by scientists other than the reporter of the case, and discussions among experts. Each case can individually be assessed and receive a final causality score for each suspected synthetic drug, herb, or dietary supplement. The updated RUCAM is improved by providing a better definition of the elements to take into consideration and more accuracy in data elements to assist the exclusion of alternative causes.
